# Integrative analysis of gut microbiome and metabolites revealed novel mechanisms of intestinal *Salmonella* carriage in chicken

**DOI:** 10.1038/s41598-020-60892-9

**Published:** 2020-03-16

**Authors:** Khin K. Z. Mon, Yuhua Zhu, Ganrea Chanthavixay, Colin Kern, Huaijun Zhou

**Affiliations:** 1 Department of Animal Science, University of California, Davis, CA 95616 USA; 2 State Key Laboratory of Animal Science, China Agricultural University, Beijing, China

**Keywords:** Microbiome, Pathogens

## Abstract

Intestinal carriage of *Salmonella* Enteritidis (SE) in the chicken host serves as a reservoir for transmission of *Salmonella* to humans through the consumption of poultry products. The aim of the current study was to examine the three-way interaction that occurred between host metabolites, resident gut microbiota and *Salmonella* following inoculation of SE in two-week-old layer chicks. Our results revealed an overall alteration in gut microbiome and metabolites in association with SE infection. Enriched colonization by different microbial members throughout the course of experimental infection highlighted significant fluctuation in the intestinal microbial community in response to *Salmonella* infection. As changes in community membership occurred, there was also subsequent impact on differential regulation of interlinked predicted functional activities within the intestinal environment dictated by *Salmonella*-commensal interaction. Alteration in the overall microbial community following infection also has a ripple effect on the host regulation of cecum-associated metabolic networks. The findings showed that there was differential regulation in many of the metabolites in association with SE colonization in chickens. Perturbation in metabolic pathways related to arginine and proline metabolism as well as TCA cycle was most prominently detected. Taken together, the present findings provided a starting point in understanding the effect of intestinal *Salmonella* carriage on the microbiome and metabolome of developing young layer chicks.

## Introduction

*Salmonella enterica* subsp. *enterica* serovar Enteritidis (SE) infection in the chicken host has a significant impact on the poultry industry as it serves as a source of contamination not only to other chickens in co-housing facilities but also due to its high zoonotic potential for the human population through consumption of contaminated food. In fact, CDC reported over half a billion eggs were recalled in 2010 due to a nationwide outbreak of human foodborne salmonellosis caused by Salmonella Enteritidis contamination in shell eggs (https://www.cdc.gov/salmonella/2010/shell-eggs-12-2-10.html). In addition, most of the *Salmonella* serovars (more than 2,500 serotypes classified) are not host-restricted and have the capability to colonize a wide variety of the animal species, which make it even more difficult to detect and eradicate the pathogen^1^. In the chicken host, depending on the immune-competence level of the host as well as infecting *Salmonella* serovars, three potential disease outcome can result: acute/fatal, chronic salmonellosis or bacterial clearance^2,3^. One of the most prevalent causes of food-borne associated illness in human is to *Salmonella* Enteritidis^4,5^.

The gut of newly hatched chicks is a relatively sterile environment, which provides ample opportunity for the incoming pathogen or other microorganisms to colonize and expand freely in the gut^6,7^. During the early post-hatch period, chicks are exposed to external factors such as food, water, and environment that begin the transitional developmental stage of the gut microbiota, which undergoes dynamic changes with increased diversity and complexity as host ages^8–10^. Exposure to SE during the early post-hatch period in chickens is critically detrimental to the overall development of the gut microbiota, with reported observations of reduction in microbial diversity as well as expansion in pathogen related members of the microbial community^6,11^. During the first month of life, the cecum microbial community of the chicken host undergoes dramatic fluctuation with species succession and changes^12^. Mature and strong presence of the core gut microbiota community is a key to developing stable functional gastrointestinal tracts with the ability to confer protection against potential pathogen colonization. Transfer of mature and stable gut microbiota from adult chickens to newly hatched chicks also has suggested its effect on improving colonization resistance against SE^7^. To our knowledge, the potential ability of the transitional phase in developing resident gut microbiota of two-week old chicks to confer protection against SE colonization in the gut has not yet been explored.

Intestinal carriage of *Salmonella* Enteritidis in the poultry host does not cause substantive gastrointestinal disease and is asymptomatic^13^. In order for pathogens like SE to colonize, survive and persist in the hostile intestinal environment of eukaryotic hosts with competing commensal microbiomes, it will either have to alter the host molecular & cellular functions or the host will have to reprogram its tolerance strategies towards the pathogen^14^. Maintenance of the overall gut homeostasis environment requires highly comprehensive interplays between the host, microbial communities and their metabolites^15^. Composition of gut-associated metabolites can be re-shaped by the interaction that occurred between the host, *Salmonella*, and resident gut microflora during the infection^16^. Therefore, integrative analysis of intestinal microbiota and gut-associated metabolomic response during the course of SE infection will provide a more in-depth understanding of the three-way interaction among the host, *Salmonella* and microbial community. This will lead to the development of novel prevention and control strategies of SE infection in poultry production. The aim of the current study was to investigate and characterize the effect that SE infection has on the resident gut microbiome, microbial functional activities and the metabolome in young layer chicken hosts.

## Results

### Colonization and persistence of *Salmonella* Enteritidis in two-week old layer chicks

**Figure 1:**
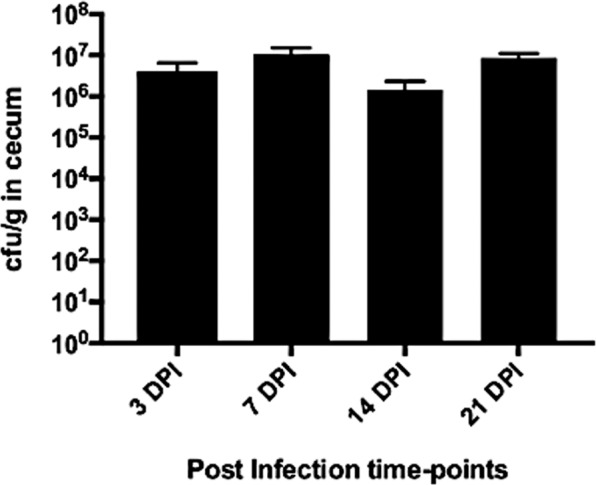
Persistent high cecal-colonization number of SE across four post-infection time-points. Bacterial load recovered from cecal content were plotted and represented as geometric mean + standard errors. All data from two separate trials were combined and represented in the graph. Approximately 33 samples contributed to the means at each timepoints.

After oral inoculation of two-week-old layer chicks with SE, no clinical signs were observed in the infected chicks. At the early infection time-point of 3 dpi (days post-infection), successful colonization of SE was detected in cecal content at 4.1 × 10^6^ cfu/g. Subsequently, the bacterial burden in the cecum of the infected chicks remained at a similar level throughout the experiment until 21 dpi (Fig. 1). At all measured post-infection time points, SE colonization was confined to the cecum with no bacterial load recovered from the systemic organs of spleen and liver. The geometric means of the bacterial loads recovered from the cecum were combined from two separate animal trials as there was no significant difference detected in the dataset between two trials.

### Effect of SE persistent colonization on the resident microbial population

**Figure 2:**
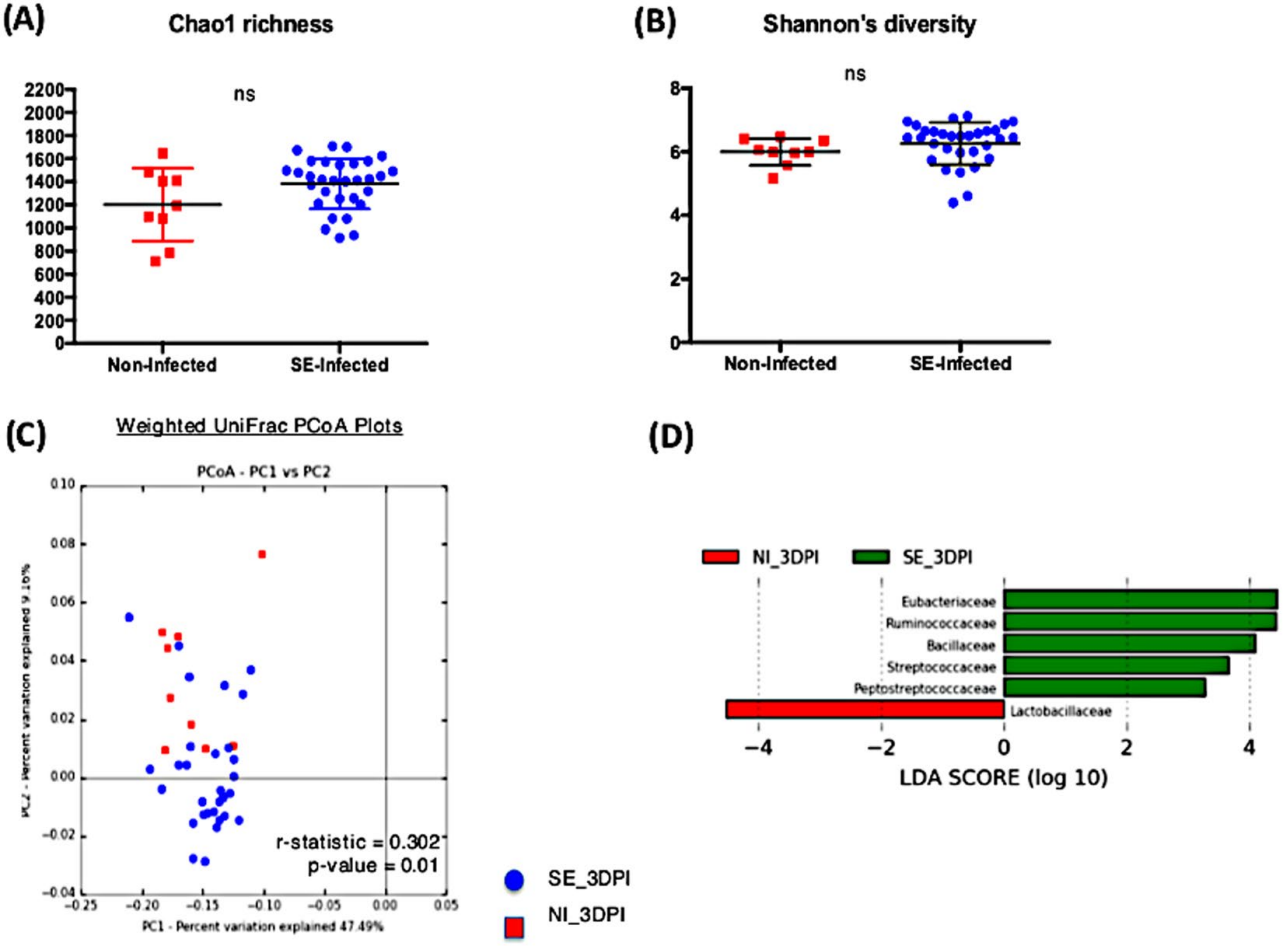
Comparison analysis of cecum microbiota profile at 3dpi: (**A,B**) alpha diversity metrics, (**C**) beta diversity, and (**D**) LEfSe analysis. (**A**) Chao1 richness estimate, (**B**) Shannon’s diversity index, (**C**) Principal coordinates analysis (PCoA) performed with weighted UniFrac distances showed no clear separation pattern between two groups. (**D**) Histogram of the Linear Discriminant Analysis (LDA) score computed for differentially abundant taxa (family level) with cut-off LDA score >2.0. Negative LDA score (red) highlight the enriched taxa in non-infected chicks and positive LDA score (green) are abundant taxa in SE-infected chicks.

**Figure 3:**
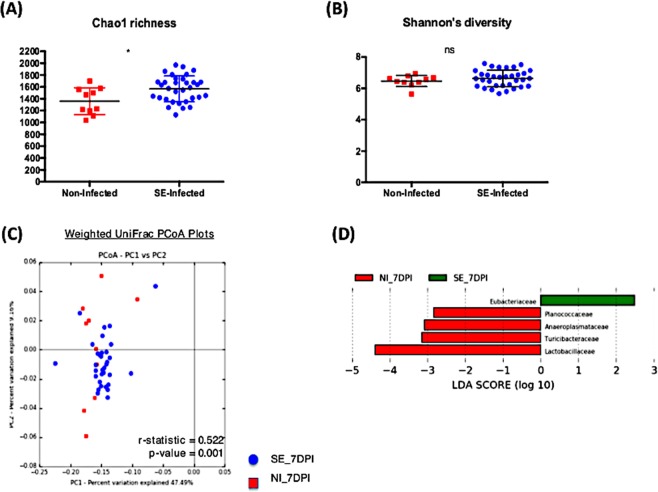
Comparison analysis of cecum microbiota profile at 7dpi: (**A,B**) alpha diversity metrics, (**C**) beta diversity, and (**D**) LEfSe analysis. (**A**) Chao1 richness estimate, (**B**) Shannon’s diversity index, (**C**) Principal coordinates analysis (PCoA) performed with weighted UniFrac distances showed no clear separation pattern between two groups. (**D**) Histogram of the Linear Discriminant Analysis (LDA) score computed for differentially abundant taxa (family level) with cut-off LDA score >2.0. Negative LDA score (red) highlight the enriched taxa in non-infected chicks and positive LDA score (green) are abundant taxa in SE-infected chicks.

**Figure 4:**
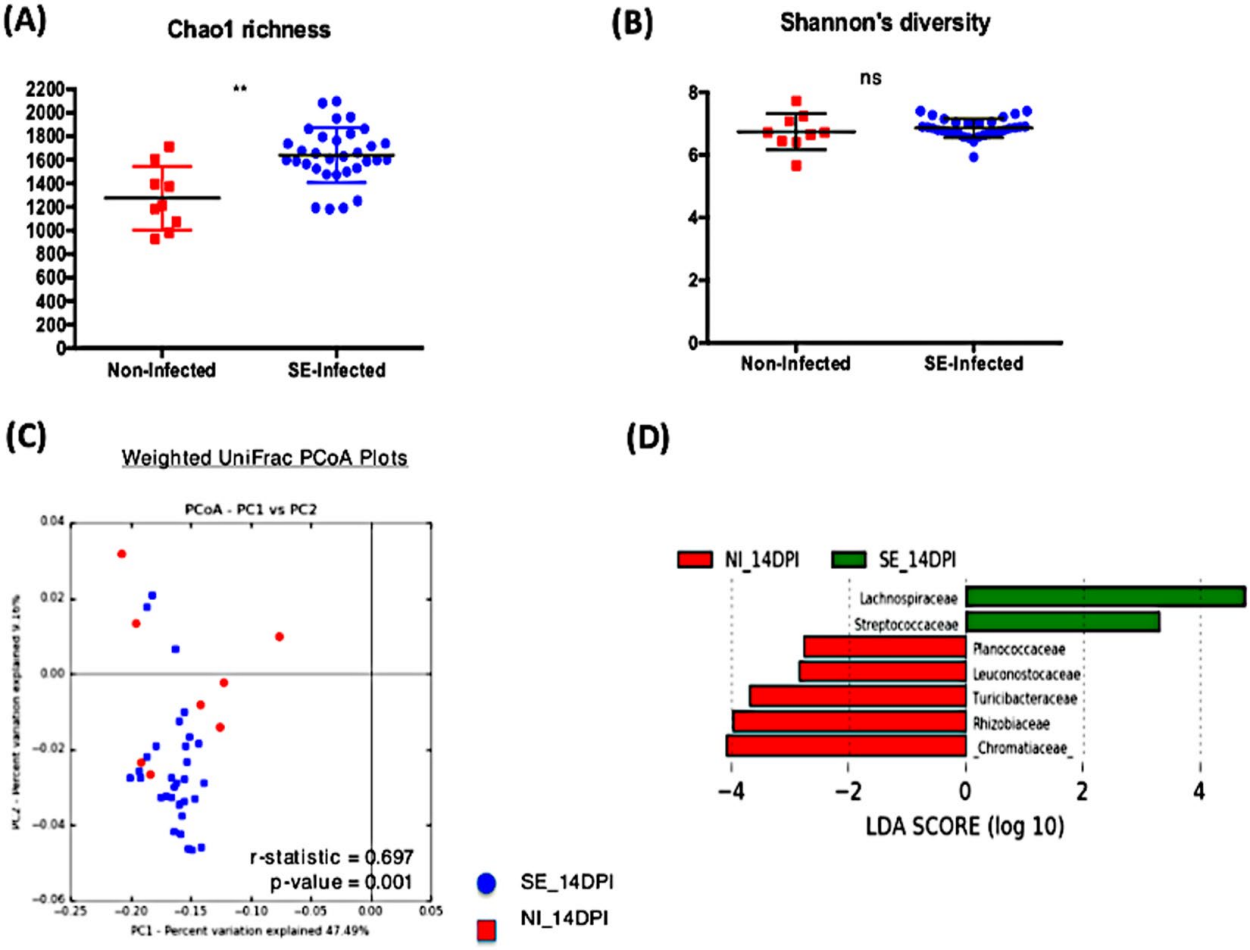
Comparison analysis of cecum microbiota profile at 14dpi: (**A,B**) alpha diversity metrics, (**C**) beta diversity, and (**D**) LEfSe analysis. (**A**) Chao1 richness estimate, (**B**) Shannon’s diversity index, (**C**) Principal coordinates analysis (PCoA) performed with weighted UniFrac distances showed no clear separation pattern between two groups. (**D**) Histogram of the Linear Discriminant Analysis (LDA) score computed for differentially abundant taxa (family level) with cut-off LDA score >2.0. Negative LDA score (red) highlight the enriched taxa in non-infected chicks and positive LDA score (green) are abundant taxa in SE-infected chicks.

**Figure 5:**
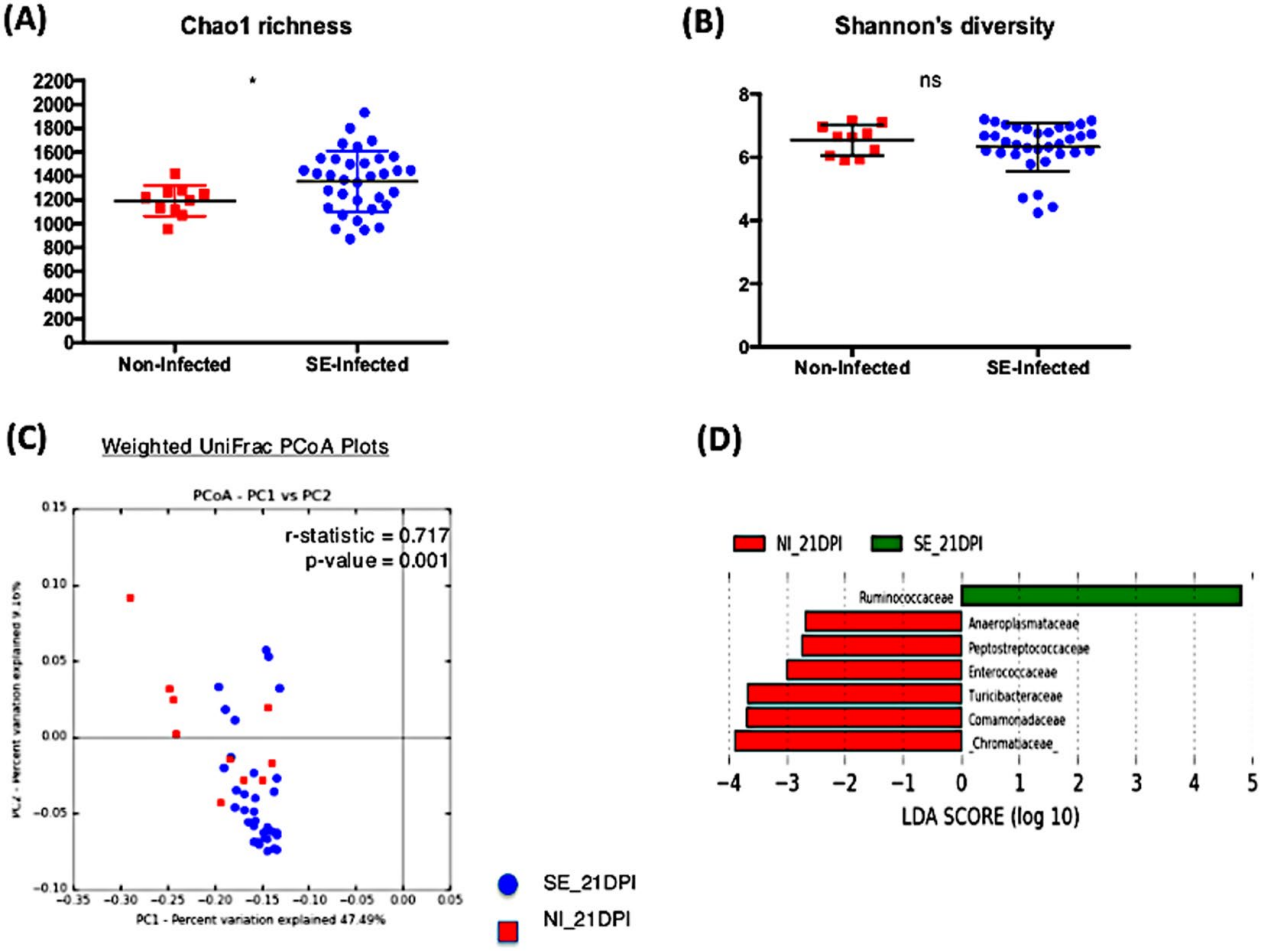
Comparison analysis of cecum microbiota profile at 21dpi: (**A,B**) alpha diversity metrics, (**C**) beta diversity, and (**D**) LEfSe analysis. (**A**) Chao1 richness estimate, (**B**) Shannon’s diversity index, (**C**) Principal coordinates analysis (PCoA) performed with weighted UniFrac distances showed no clear separation pattern between two groups. (**D**) Histogram of the Linear Discriminant Analysis (LDA) score computed for differentially abundant taxa (family level) with cut-off LDA score >2.0. Negative LDA score (red) highlight the enriched taxa in non-infected chicks and positive LDA score (green) are abundant taxa in SE-infected chicks.

There were a total of 168 samples combined from two independent animal trials with 130 SE infected chicks and 38 non-infected chicks across four time-points. While analyzing the microbiome composition, rarefaction (a subsampling technique) was performed which randomly subsampled each sample to an equal count and assumes that the count of observations for each sample is equal (this addresses unequal sequencing depth problem between samples). This allowed the contrast of the microbiome ecosystem independent of the differences in sequencing depth or sample size proportion between the treatment groups. From the Illumina MiSeq dataset, a total of 2,941,667 reads were generated with 59,762 different Operational Taxonomic Unit (OTUs) identified. Alpha diversity metric, both Chao1 richness and Shannon’s diversity index, were measured between the non-infected and SE-infected groups across all time points (Figs. 2A,B, 3A,B, 4A,B, 5A,B). Comparing the Shannon’s diversity between the two groups at each time point showed no significant differences across all infection time points. On the other hand, the Chao1 richness measurement, which reflects how many different taxa are present in samples, indicated a significant increase in the SE-infected group compared to the non-infected group at all time points except 3dpi. Principle component analysis using beta-diversity with weighted UniFrac measured the phylogenetic similarities shared between the communities. Separations in the group clustering patterns on the PCoA plot (with corresponding R value reported) over the three week time course of infection indicated that more visible separation of clustering between the two groups was observed at later timepoints of infection. At 3 dpi, there was no clear separation in the clustering between the two groups (r = 0.302, p = 0.01). However as the infection time progressed, a separation between two groups became more apparent with a significant increase in r value at 7 dpi (r = 0.522, p = 0.001), 14 dpi (r = 0.697, p = 0.001) and 21 dpi (r = 0.717, p = 0.001) (Figs. 2C, 3C, 4C, 5C). To identify differentially abundant taxonomic features at the family level, the linear discriminant analysis (LDA) together with effect size measurement (LEfSe) method was utilized. LDA score provided by LEfSe identifies the members of the microbial community that were present at a different level between non-infected samples and SE-infected samples with Kruskal-Wallis sum rank test. Positive LDA scores signified increased abundance of taxonomic features in SE-infected samples while negative LDA scores represented the microbial biomarkers that were enriched in non-infected samples (Figs. 2D, 3D, 4D, 5D). Enrichment of the *Lactobacillaceae* family was identified in non-infected chicks, whereas *Eubacteriaceae*, *Ruminococcaceae*, *Bacillaceae*, *Streptococcaceae*, and *Peptostreptococcaceae* were significantly enriched in the SE-infected chicks at 3 dpi. As the SE infection progressed, a reduced number of enriched discriminative microorganisms in the infected host was identified compared to the non-infected host. Only members belonging to the *Eubacteriaceae* family were significantly enriched in the SE-infected group at 7 dpi. Once again, *Lactobacillaceae* were significantly enriched in the non-infected group along with *Planococcaceae*, *Anaeroplasmataceae*, and *Turicibacteraceae* at 7dpi. For SE-infected chicks at 14dpi, *Lachnospiraceae* and *Streptococcaceae* were significantly enriched whereas *Planococcaceae, Leuconostocaceae, Turicibacteraceae, Rhizobiaceae*, and *Chromatiaceae* were overrepresented in the non-infected host. Overrepresentations of six groups were identified at 21 dpi in the non-infected host, which included the *Anaeroplasmataceae, Peptostreptococcaceae, Enterococcaceae, Turicibacteraceae, Comamonadaceae*, and *Chromatiaceae* family. The *Ruminococcaceae* family was the only group identified to be highly enriched in the SE-infected chicks at 21dpi.

### Predictive microbial functions based on 16S rdna data (PICRUSt analysis)

**Figure 6:**
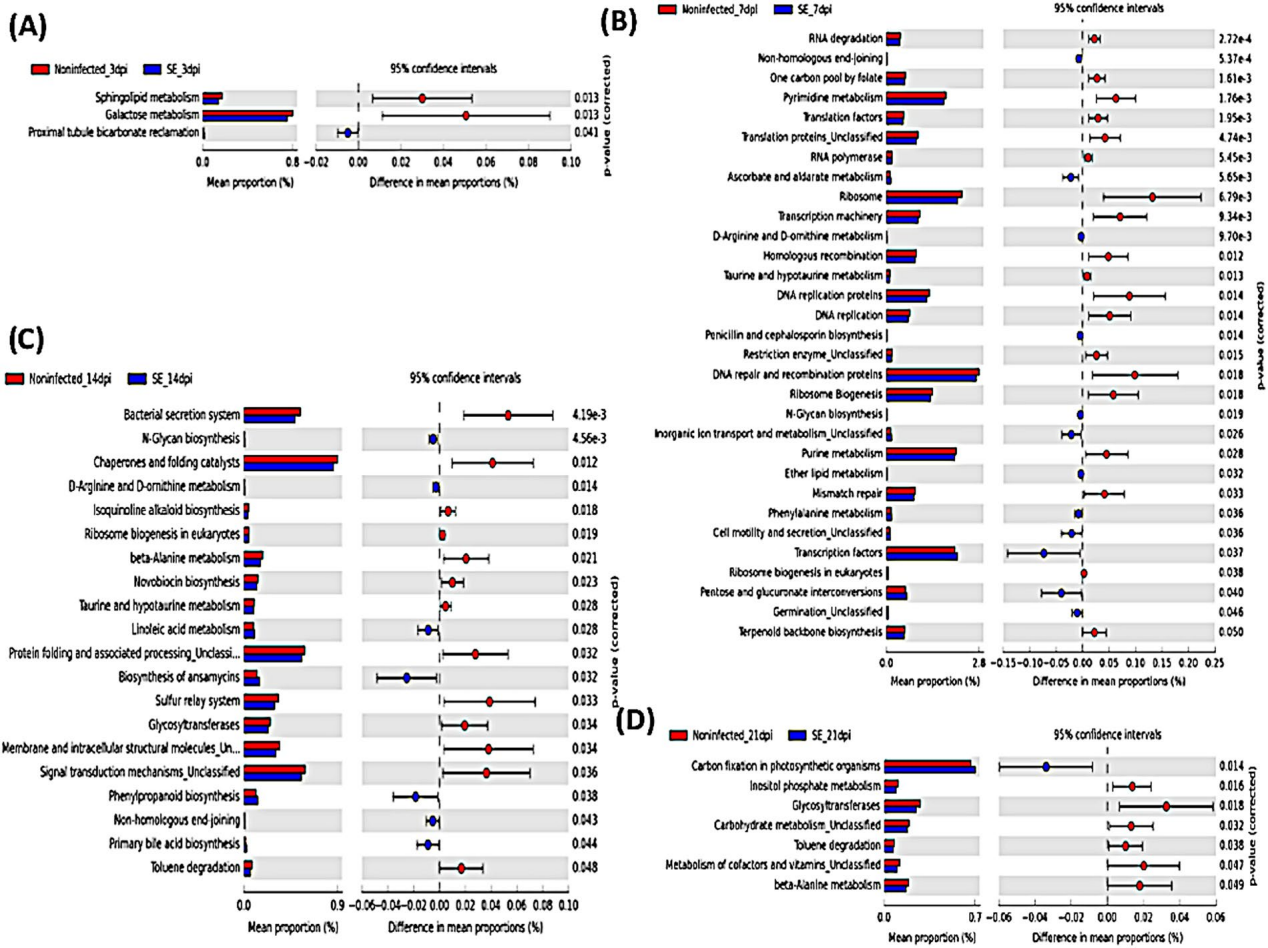
PICRUSt metagenome inference analysis based on 16S rRNA dataset: (**A**) 3dpi, (**B**) 7dpi, (**C**) 14dpi, and (**D**) 21dpi. (**A–D**) Prediction of significant KEGG pathways (level 3) that were differentially regulated in SE-infected group compared to non-infected group (p < 0.05). Mean proportion of functional pathways is illustrated with bar plots and dot plots indicate the differences in mean proportions between two groups based on p-values obtained from two-sided Welch’s t-test.

The Phylogenetic Investigation of Communities by Reconstruction of Unobserved States (PICRUSt) tool was applied to infer intestinal microbial functions in association with SE infection. Based on the microbiome profile obtained from 16S rRNA data and the available reference genome database, PICRUSt predicts the functional composition of the metagenome in host-associated communities^17^. Differentially abundant functional features between the infected and the non-infected groups were compared and analyzed by Statistical Analysis of Metagenomic Profiles (STAMP)^18^. Predicted microbial functions were then aligned to the Kyoto Encyclopedia of Genes and Genomes (KEGG), level 3, to identify significant differences (p < 0.05) in biological processes and pathways (Fig. 6A–D). Functional pathways associated with human diseases were filtered out from the analysis. Only a few of the predicted functional pathways were differentially regulated at 3dpi and 21dpi timepoints with significant downregulation in categories mostly related to metabolism (sphingolipid, galactose, inositol phosphate, carbohydrate, vitamins & cofactor, beta-alanine) in the SE-infected group. There was more fluctuation in predicted microbial functional pathways at 7dpi and 14dpi. A total of thirty-one differentially abundant functional pathways were predicted at 7dpi (12 enriched and 19 decreased abundance in the infected chicks compared to the non-infected). Specifically, functional pathways that were related to genetic information processing (RNA degradation, ribosome, DNA replication, ribosome biogenesis, mismatch repair) as well as nucleotide metabolism (purine, pyrimidine) were significantly downregulated in the SE-infected group at 7dpi. At 14 dpi, 20 predicted KEGG pathways were differentially represented with seven of them enriched, while thirteen were downregulated in association with SE infection. Functional genes associated mostly with general cellular metabolism & biosynthesis and environmental processing and signaling (bacterial secretion system, membrane and intracellular structural molecules, signal transduction mechanisms) were more abundant in the non-infected chicks.

### Effect of SE persistent colonization on cecum metabolites profile

For metabolomic profiling, cecum samples were randomly selected from two independent trials with equal sample sizes from both treatment groups across four timepoints (with the exception of one sample at 14 dpi that failed the mass spectrometry run and was omitted from further data analysis). A total of 435 metabolites (191 identified and 244 unknown) from 39 samples were identified from cecal content using Gas Chromatography-Time-of-Flight Mass Spectrometry (GC-TOF MS). The metabolites datasets were input into MetaboAnalyst^19–24^, a web-based analytical pipeline for data processing, normalization, statistical analysis and metabolic pathway analysis. Both univariate and multivariate analyses were performed on the metabolites datasets to compare and identify the distinguishing features that differentiate the gut-associated metabolites of non-infected chicks and SE-infected chicks across four time-points. The univariate analysis with volcano plot method (comparing the fold change to statistical significance) identified significant accumulation of metabolites in the SE-infected group compared to the non-infected group at different timepoints (Table 1). At 3 dpi, a total of 6 significant metabolites were found with 4 identified and 2 unknown metabolites. A total of 78 significant metabolites (33 known & 45 unknown) were identified at 7dpi, the most across four time points. Out of the 33 identified metabolites, 8 metabolites were significantly reduced while 25 metabolites were significantly increased in the SE-infected group. There was a total of 3 differentially expressed metabolites found at 14 dpi (1 known & 2 unknown) with up-regulation of 1 annotated metabolite in the SE-infected group. At 21 dpi, 21 metabolites were significantly altered with 9 known and 12 unknown metabolites. Only 3 identified metabolites were found accumulated while 6 identified metabolites found to be reduced in the SE-infected group compared to the non-infected group.

**Table 1:** List of statistically significant biomarker metabolites selected from combined analysis of volcano plot analysis (fold change 1.5 & FDR adjusted p-value ≤ 0.1 from t-test), PLS-DA (VIP score >1.0), and SAM (q-value ≤ 0.1) with its respective statistical values.

DPI	Metabolites	Volcano plot			SAM	PLS-DA
		FC	log2(FC)	FDR adjusted p-value	q-value	VIP score
3 DPI	hexadecylglycerol NIST	0.353	−1.501	0.051	0.069	2.321
	Isoribose	0.072	−3.802	0.073	0.080	2.219
	octadecylglycerol	0.202	−2.306	0.073	0.080	2.215
	5-aminovaleric acid	0.031	−5.016	0.077	0.091	2.194
7 DPI	citric acid	8.581	3.101	0.009	0.018	1.817
	uric acid	5.561	2.476	0.005	0.016	1.866
	adipic acid	3.203	1.679	0.046	0.037	1.608
	2-ketoisocaproic acid	2.844	1.508	0.028	0.027	1.712
	O-acetylserine	0.621	−0.688	0.046	0.037	1.609
	3,4-dihydroxycinnamic acid	0.597	−0.745	0.019	0.022	1.757
	2-hydroxy-2-methylbutanoic acid	0.596	−0.746	0.068	0.049	1.552
	homocystine	0.523	−0.934	0.046	0.037	1.613
	pyridoxine	0.401	−1.320	0.028	0.027	1.705
	alanine-alanine	0.386	−1.373	0.074	0.052	1.532
	xylonolactone NIST	0.383	−1.384	0.079	0.056	1.509
	galactinol	0.379	−1.399	0.064	0.047	1.560
	glutamine	0.331	−1.597	0.110	0.076	1.434
	2-hydroxyglutaric acid	0.314	−1.671	0.104	0.071	1.469
	D-erythro-sphingosine	0.306	−1.710	0.074	0.052	1.523
	Daidzein	0.271	−1.882	0.051	0.041	1.586
	3-hydroxybenzoic acid	0.251	−1.995	0.030	0.028	1.694
	3,4-dihydroxybenzoic acid	0.227	−2.141	0.045	0.036	1.629
	2-isopropylmalic acid	0.201	−2.315	0.031	0.028	1.693
	nicotinamide	0.196	−2.354	0.005	0.016	1.856
	putrescine	0.179	−2.484	0.005	0.016	1.868
	adenosine	0.151	−2.727	0.061	0.045	1.604
	5-hydroxy-3-indoleacetic acid	0.140	−2.841	0.076	0.054	1.528
	4-hydroxybenzoate	0.123	−3.029	0.046	0.037	1.614
	Inosine	0.104	−3.268	0.038	0.030	1.652
	beta-glutamic acid	0.095	−3.394	0.104	0.071	1.458
	Isoribose	0.077	−3.694	0.018	0.022	1.764
	3,4-dihydroxyphenylacetic acid	0.050	−4.309	0.110	0.076	1.428
	5-aminovaleric acid	0.022	−5.481	0.000	0.014	1.962
14 DPI	fructose-6-phosphate	0.328	−1.608	0.109	0.091	2.185
21 DPI	2-hydroxyvaleric acid	1.805	0.852	0.052	0.049	1.999
	2-hydroxy-2-methylbutanoic acid	1.769	0.823	0.112	0.090	1.932
	4-hydroxyphenylacetic acid	6.667	2.737	0.136	0.098	1.804
	octadecanol	2.874	1.523	0.136	0.099	1.798
	pentadecanoic acid	2.857	1.515	0.136	0.097	1.845
	O-acetylserine	3.161	1.660	0.140	0.103	1.798

**Figure 7:**
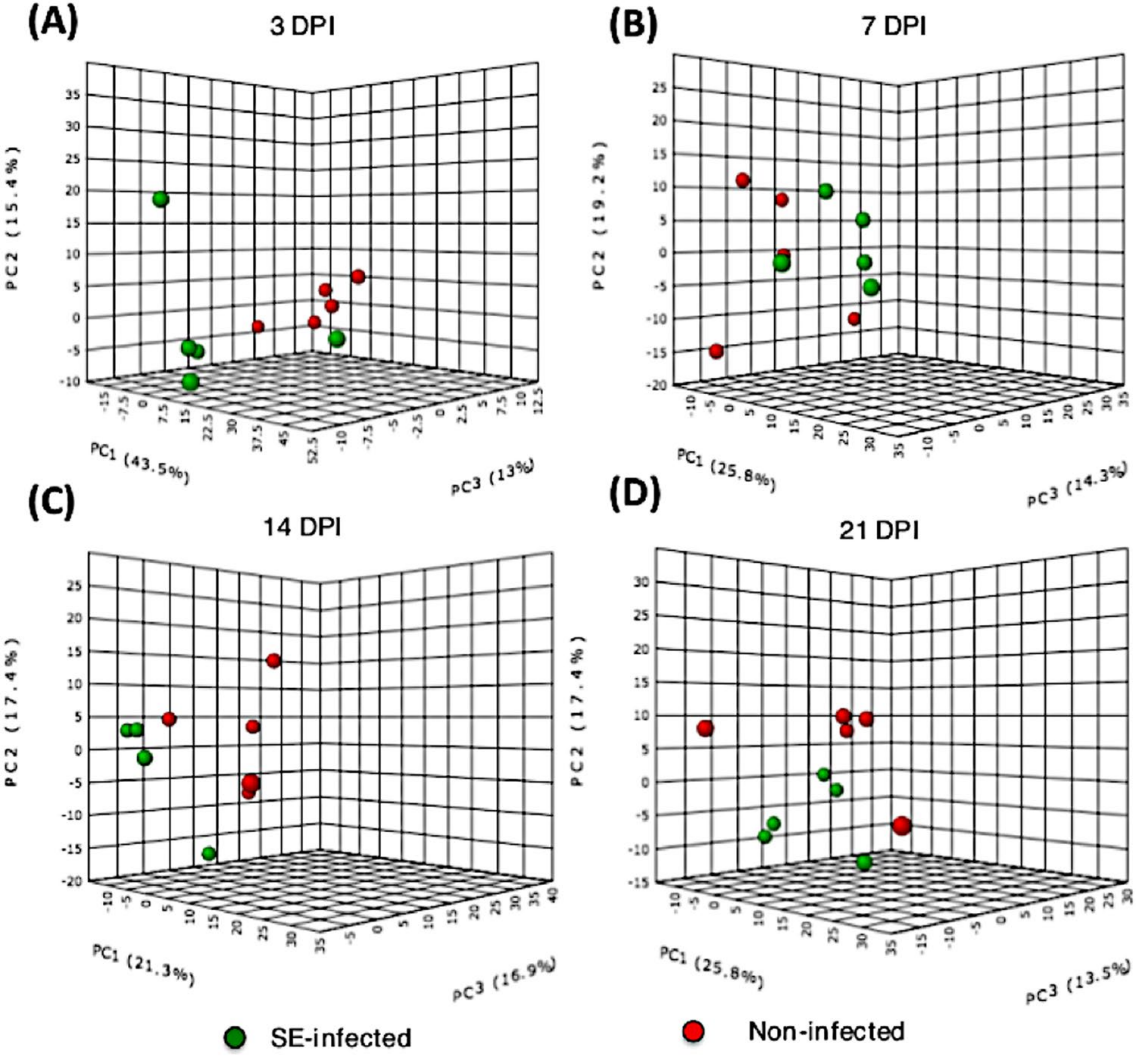
Unsupervised pattern recognition method, 3D Principal Component Analysis (PCA) plot was generated with normalized sample peak intensities across four time-points to detect the intrinsic cluster between two treatment groups as well as to identify possible outliers within group: (**A**) 3dpi, (**B**) 7dpi, (**C**) 14dpi, and (**D**) 21dpi.

**Figure 8:**
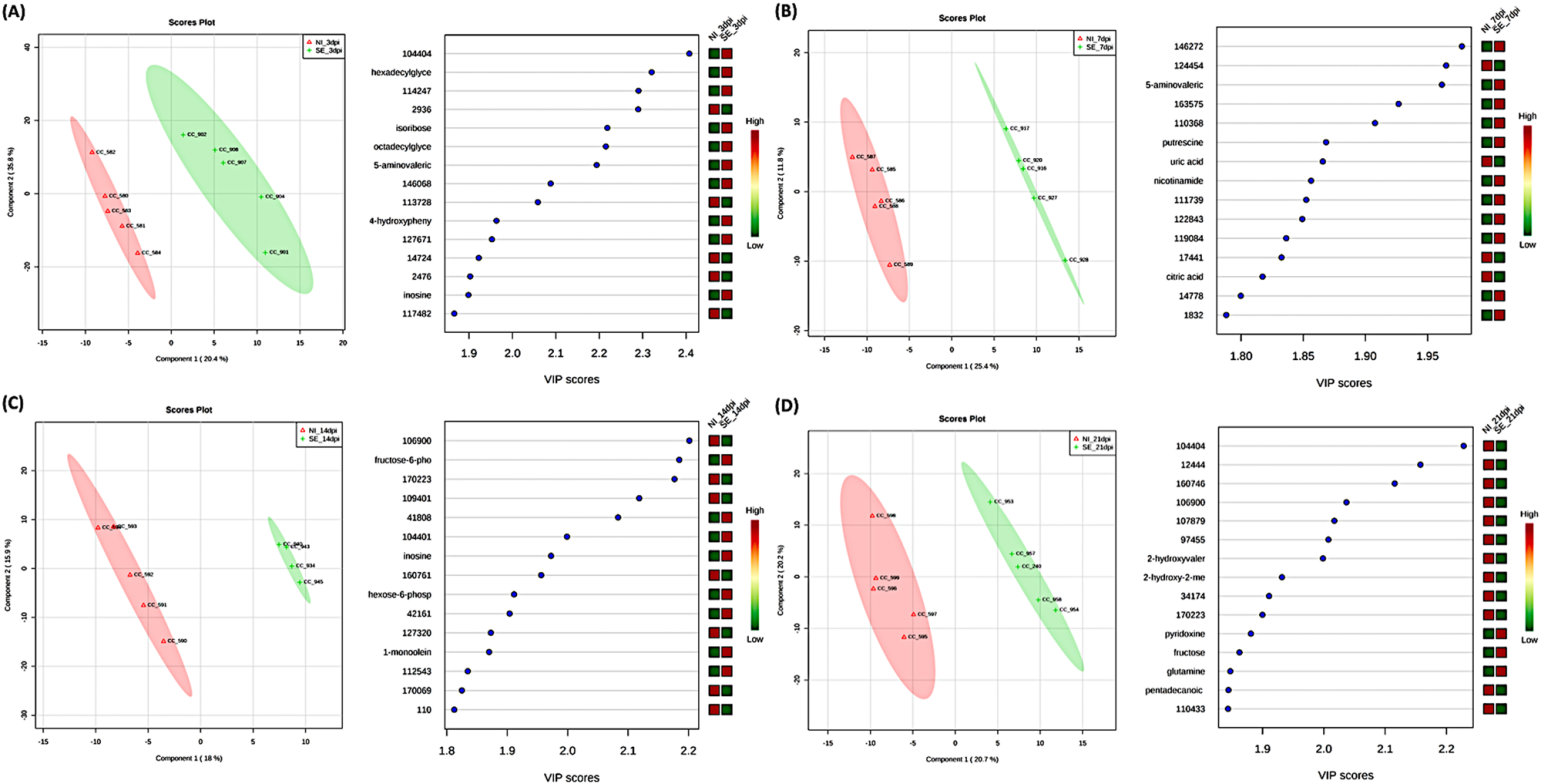
PLS-DA score plots for comparisons of the metabolites profiles in the SE-infected (green) and the non-infected (red) groups with top 15 important metabolites selected on the basis of VIP score. High VIP score indicates greater contribution of the metabolites to the group separation. (**A**) 3dpi, (**B**) 7dpi, (**C**) 14dpi, and (**D**) 21dpi.

**Figure 9:**
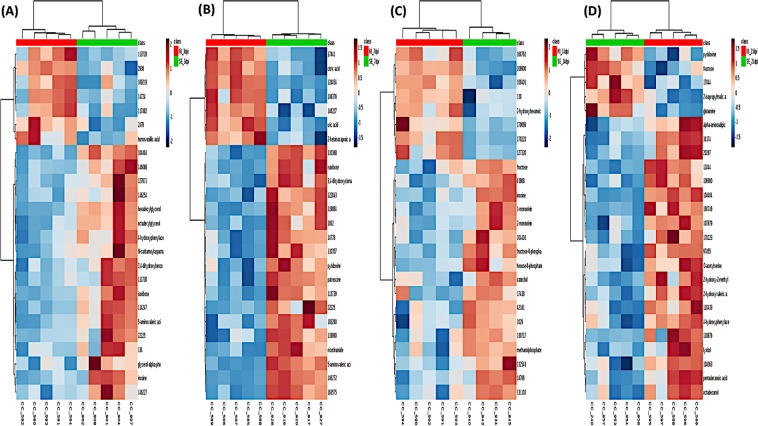
Heat map of the top 25 most significant metabolites from student t-test’s analysis that distinguished between the non-infected and the SE-infected groups (**A**) 3dpi, (**B**) 7dpi, (**C**) 14dpi, and (**D**) 21dpi.

For multivariate analysis, both PCA and PLS-DA were performed to identify discriminant features associated with SE infection. Three-dimensional PCA followed by an unsupervised pattern recognition method was first used to detect the intrinsic clusters and to detect possible outliers. The result revealed that the metabolite profile between the two groups exhibited an ambiguous separation pattern with some degree of overlap in clustering, as seenin the PCA plots (Fig. 7). To further examine the differential abundance pattern of metabolites and to identify the potential biomarkers contributing to group separation, PLS-DA^24^, a supervised method, was performed. A much better group separation pattern that distinguishes the non-infected and SE-infected groups was seen at all four time-points in the two-dimensional score plot generated by PLS-DA (Fig. 8A–D). Variance in the data among the five component measurements was derived and PLS-DA score plots showing the variance in component 1 and component 2 were generated, respectively. To make an assessment on the accuracy of the PLS-DA predictive model, as well as the statistical significance of the analysis, leave-one-out cross validation was performed with reported accuracy rate, R^2^ and Q^2^ value for each of the five components at all four time points (Table 2). Across all four time-points, the PLS-DA model on the current data had consistently high accuracy rates of >75%, R^2^ value at ~0.99, and Q^2^ scores >0.5 (at 7dpi) and close to 0.5 (3dpi, 21 dpi) except at 14 dpi where Q^2^ <0.5, suggesting that the PLS-DA model was robust. To identify metabolites that contribute most significantly to a separation between the two groups in the PLS-DA plot, Variable Importance in Projection (VIP) scores were analyzed where high VIP scores indicated a greater contribution of the metabolites to the group separation. The top 15 metabolites at each time-point with their respective VIP scores are shown in (Fig. 8A–D). The Student’s t-test was used to identify the top 25 metabolites that were differentially altered between the non-infected and the SE-infected groups, which were visualized using a heat map (Fig. 9A–D). This analysis suggested that the majority of the metabolites were highly abundant in the SE-infected group at first three time points of infection (3 dpi, 7 dpi, and 14 dpi). However, at 21 dpi a reversed pattern in metabolite abundance was found with only a few metabolites being more abundant in the SE-infected group compared to the non-infected group.

**Table 2:** Result of leave-one-out cross validation for PLS-DA model with reported accuracy rate, R^2^ and Q^2^ value for each of the five components across four timepoints.

DPI	Measure	1 comps	2 comps	3 comps	4 comps	5 comps
3 DPI	Accuracy	0.6	0.8	0.8	0.8	0.8
	R2	0.852	0.975	0.995	0.999	0.999
	Q2	−0.047	0.430	0.499	0.498	0.450*
7 DPI	Accuracy	1.0	1.0	1.0	1.0	1.0
	R2	0.956	0.995	0.999	1.0	1.0
	Q2	0.703	0.778	0.783	0.789	0.790*
14 DPI	Accuracy	0.778	0.667	0.778	0.778	0.778
	R2	0.952	0.998	1.0	1.0	1.0
	Q2	0.244	0.313	0.336	0.342	0.342*
21 DPI	Accuracy	0.8	0.8	0.8	0.7	0.7
	R2	0.918	0.981	0.998	1.0	1.0
	Q2	0.412	0.465*	0.458	0.451	0.448

Accuracy rate >75%, R^2^ value = 1, Q^2^ scores >0.5 confirm the significant & predictive power of the PLS-DA model.

### Differential regulation of metabolic pathway analysis associated with SE infection

**Figure 10:**
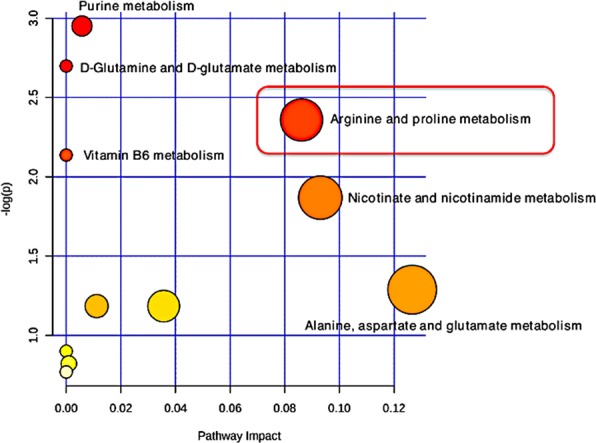
Metabolic pathway analysis performed with up-regulated biomarkers metabolites from all four time-points that matched the Gallus gallus (Chicken) database. Larger circles, higher and closer to Y-axis showed higher impact of the pathway and color ranging from yellow to red mean metabolites from input list are involved in the pathway with different level of significance (Red = higher significance). Arginine and proline metabolism most significantly enriched with –log p-value >2 and pathway impact >0.06.

**Figure 11:**
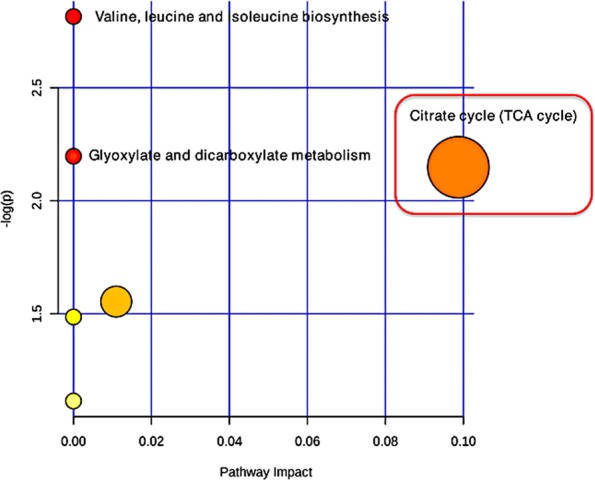
Metabolic pathway analysis performed with down-regulated biomarkers metabolites from all four time-points that matched the Gallus gallus (Chicken) database. Larger circles, higher and closer to Y-axis showed higher impact of the pathway and color ranging from yellow to red mean metabolites from input list are involved in the pathway with different level of significance (Red = higher significance). Citrate cycle (TCA cycle) was most significantly perturbed with –log p-value >2 and pathway impact >0.06.

The Significance Analysis for Microarrays (SAM) method^24^ was utilized to identify the most discriminant biomarker metabolites associated with the SE infected state, using q-value ≤ 0.1. Collectively, significant metabolites identified from the univariate analysis, PLS-DA and SAM were then screened and selected through a statistical significance criteria of FDR adjusted p-value <0.1, fold change threshold >1.5, q-value <0.1, and PLS-DA VIP score >1.0 for pathway analysis. A list of selected biomarker metabolites with their respective statistical values is presented in Table 2. Un-annotated metabolites were omitted from the list for further downstream pathway analysis. Based on the significant known biomarkers that matched the database, metabolic pathway enrichment analysis was performed to identify the potential pathways that were perturbed during SE infection. Up-regulation of 11 potential metabolic pathways were identified in response to SE infection (Fig. 10). Out of the 11 pathways identified, arginine and proline metabolism were the most significantly enriched with –log p-value >2 and pathway impact >0.06. Likewise, potential downregulation of metabolic pathways was determined using biomarker metabolites that were significantly reduced in the SE-infected group compared to the non-infected group. A total of six potential metabolic pathways were down-regulated in response to SE infection. Out of them citrate cycle (TCA cycle) was most significantly perturbed with –log p-value >2 and pathway impact >0.06 (Fig. 11).

## Discussion

Immediately after hatch, exposure to exogenous microorganisms in the environment begins the assembly and developing phase of the gut microbiota in the chick host^10^. The microbial community then begins to diverge and its composition (both diversity and abundance of each species) fluctuates as the chick ages^8,9^. Two-week old chicks presented a unique infection model as it helped to address the question of whether the transitional developmental stage of the gut microbiota in chicken hosts provide a protective role in gut colonization resistance against *Salmonella*. Despite high doses of SE inoculum used in the study, we didn’t observe a systemic infection phenotype in our model and bacterial colonization was exclusively localized in the cecum of the infected chicks. In the current study, the intricate interplay at the intestinal level between *Salmonella*, host and resident gut microbiota was examined through integrative analysis of the microbiome, predictive metagenome, and gut-associated metabolites.

We had previously characterized the structural changes that occur in the gut microbiota of one-day old chicks as a direct consequences of SE infection. Findings from our previous study showed that inoculum of SE in newly hatched chicks significantly altered the cecum microbiota with an overall reduction in diversity and expansion in specific members of the community: the *Enterobacteriaceae* family^11^. With the current two-week old infection model, changes in the gut microbiome community were less substantial in comparison, with an absence of a specific species dominance in the community linked to SE-infected status. This was supported by the Shannon’s diversity index data from all four post-infection timepoints (no significant difference) suggesting that distributions of major microbial members of the community between the SE-infected and non-infected groups were similar. On the other hand, a spike in species richness (Chao1 index) associated with SE infection across three time-points (7dpi, 14dpi, 21dpi) in this study indicates increased colonization by minority members of the community following the infection. In the current model, fluctuation in the microbiome community appears to occur independently without affecting the *Salmonella* colonization in the intestine. Differences in the microbial perturbation signature between the one-day old and two-week old infection model might be related to both the gut-associated immune system and the microbiota developmental stage of the host.

Metagenome functional prediction associated with the chicken gut microbiome also highlighted differential regulation in various pathways between the non-infected and the infected groups. The functional changes occurring at the earliest (3dpi) and latest (21dpi) timepoint of infection were much fewer in comparison to significant fluctuation occurring at the intermediate stages of the experimental infection timeline (7dpi and 14dpi). Lack of drastic functional gene differences at the late timepoint of SE infection (21dpi) might indicate the microbial community between the two groups adopted similar metagenomic patterns despite exhibiting taxonomic differences. Reduced activity in genetic information processing and nucleotide metabolism (purine & pyrimidine) in the SE-infected chickens might suggest a scale back in the maintenance of microbial cellular and intestinal activity at 7dpi. Functional genes associated with ribosomal activity were also more abundant in the non-infected group than the SE-infected group. Such decrease in ribosomal activity might suggest that *Salmonella* infection potentially interfere with key protein biosynthesis processes of intestinal microbes. Similarly, the taxonomic changes at 14dpi were also accompanied by an alteration in the predictive metagenomic profile of the SE-infected chicks. There was downregulation in bacterial secretion system, signal transduction mechanism and membrane intracellular structural molecules pointing towards a possible downshift in intestinal microbes’ ability for adhesion and attachment of eukaryotic cells. This may provide a plausible explanation in terms of microbial membership fluctuation within the community during the experimental time course of infection.

Persistence of *Salmonella* intestinal colonization in birds trigger the host to undergoes immunometabolic reprogramming in an effort to limit the damage caused by the *Salmonella* without affecting or reducing the bacterial load in the poultry host^14,25,26^. Overall cecum-associated metabolomic dataset as well as our unpublished RNA-seq data on the cecal tonsil from the same birds provided evidence that there are changes in host response to SE infection in terms of both global gene expression pattern and metabolites profile. The most significant numbers of differentially expressed metabolites were detected at 7dpi. Specifically, up-regulation of arginine and proline metabolism was detected in association with SE infection. Unpublished cecal tonsil RNA-seq data also showed the upregulation of arginine associated pathways at 3dpi, confirming and validating the metabolites pathway analysis. Arginine availability in the host, as well as arginine utilization strategies by the host, largely contribute to the regulation of the host defense mechanism and outcome of the disease during the infection^27^. Arginine is a common amino acid substrate that is competed for by both arginase for ornithine & proline synthesis and inducible nitric oxide synthase (iNOS) for nitric oxide production^28^. Generation of nitric oxide (NO) by iNOS during *Salmonella* infection is one of the key innate immune responses to induce inflammation as part of the host defense mechanism^29,30^. The fate of the pathogen survival and replication within the host is dependent on the regulation of iNOS expression and subsequently NO production. Inhibition or down-regulation of iNOS has been shown to enhance intracellular proliferation and survival of *Salmonella* in mice models^31,32^. NO production can be negatively regulated by the enzymatic action of arginase through its competition for the common substrate, arginine. In our current model, arginine availability in the host appeared to be channeled towards the enzymatic action of arginase rather than iNOS to produce ornithine, which in turn serves as a precursor for the synthesis of proline, thereby essentially suppressing NO production level. Therefore, we speculate that up-regulation of arginase-associated pathways (arginine and proline metabolism) in the current model could have been part of the host metabolic adjustment strategies to dampen the intestinal inflammation during *Salmonella* infection. Consequently, the resulting metabolic adjustment of the host could offer protection for *Salmonella* colonization in the otherwise hostile environment of the gut.

In addition, the analysis of the metabolites revealed reduced citrate (TCA) cycle activity in the SE-infected group compared to the non-infected group. Inhibition or decreased activity of TCA was supported by a decreased amount of citrate acid metabolites in the cecal content of the infected chicks at 7dpi in our current study. Decreased TCA cycle activity revealed a change in host cellular energy metabolism during *Salmonella* infection. The exact mechanism by which the reduction of host TCA cycle activity is related in response to *Salmonella* infection in the gut is unknown. However, there had been some insights gained from studies related to the innate immunity metabolic reprogramming strategies. Upon activation by the invading pathogen components like lipopolysaccharide (LPS) from gram-negative bacteria, innate immune cells have been reported to undergo a metabolic switch from TCA cycle to aerobic glycolysis which results in overall decreased TCA cycle activity^33^. Therefore, innate immune activation triggered by LPS stimuli from SE might have contributed to the alteration in host metabolic response during SE infection.

In summary, a microbial shift in two-week old SE-infected hosts was directed towards the enrichment of non-specific minority members of the community in the cecum. As changes in microbial community membership occurred, there was subsequent impact on the differential regulation of the inferred metagenome functional activities of the intestinal microbes. Metabolic adjustment strategies adapted by the host in response to SE infection also resulted in significant alteration in cecum-associated metabolites and its associated pathways. Taken together, the current study provided an important novel insight into the gut microbiome’s and metabolites’ contribution to intestinal *Salmonella* carriage in chicken. Furthermore, these results have laid a solid foundation for further investigation of the underlying molecular mechanism of SE colonization and persistence in the chicken.

## Materials and Methods

### Animal experiments

The highly inbred genetic line UCD003, layer chicks from the University of California, Davis’s poultry farm, was utilized in the current study. Immediately following hatch, chicks were transferred to housing chambers with pine shavings on a concrete floor in a temperature-controlled environment. Chicks were housed in two separate chambers with identical environmental conditions to separate the control group from infected chicks. The chicks were given *ad libitum* access to water and non-medicated commercial feed. Before inoculation, all chicks were clocal swabbed to confirm the absence of SE. At two weeks of age, chicks were inoculated (via oral gavage) with 10^9^ colony forming units (c.f.u) of S. Enteritidis (TN2) kanamycin and carbenicillin-resistant strain (kindly provided by Dr. Andreas Baumler at University of California, Davis) while control chicks received phosphate buffered saline (PBS). After infection, the inoculum was checked to confirm the dosage amount through serial dilution plating method. At four time points of 3, 7, 14, and 21 days post infection (dpi), chicks were euthanized with carbon dioxide asphyxiation. Cecal contents were collected for enumeration of bacterial numbers, 16S rRNA gene sequencing and metabolite analysis. *Salmonella* Enteritidis numbers from cecal content, spleen and liver were determined by plating serial ten-fold dilution on MacConkey agar containing both kanamycin & carbenicillin antibiotics. The geometric mean of the SE bacterial counts was reported as c.f.u. per gram of the cecal content, spleen, and liver. Data collected from two separate repeated trials were combined together for microbiota analysis. For the metabolite profile, cecal content collected from five representative control chicks and five SE-infected chicks at each time point (except on 14dpi where one failed sample from SE-infected group was excluded for analysis) were submitted for Gas Chromatography-Mass Spectrometry (GC-MS) analysis. All animal experiments performed in the current study were approved by the Institutional Animal Care and Use Committees at the University of California, Davis (IACUC#19272). All experiments in this study were performed in accordance with IACUC guidelines and regulations.

### Sample preparation for 16S rRNA gene sequencing and metabolic profiling

Approximately 150 mg of cecal content harvested were first subjected to 5 minutes of bead beating step at maximum speed setting in the Bullet Blender Storm 24 (Next Advance Inc, Averill Park, NY). DNA was then isolated using the Zymo fecal DNA miniprep kit (Zymo Research, Irvine, CA) in accordance with manufacturer’s instructions. DNA concentrations were analyzed on Nanodrop and then stored at −20 °C until further use. Isolated DNA was then used as a template in PCR amplification for construction of 16S rDNA libraries. V4 hypervariable regions of the 16S rRNA gene were targeted for amplification with forward primer, F515 (5′NNNNNNNNGTGTGCCAGCMGCCGCGGTAA3′) and reverse primer, R806 (5′GGACTACHVGGGTWTCTAAT3′) (primers kindly provided by Dr. Elizabeth Maga). The V4 region was selected for its reported high classification consistency in microbial profiling studies^34,35^. The forward primer contained the linker region (GT) as well as a unique 8 base pair barcode sequence (N) for each of the individual samples to be sequenced. PCR amplification was performed in 25 µl reactions containing 12.5 µl of 2x GoTaq Green Master Mix (Promega, Madison, WI, USA), 0.5 µl of each of the forward and reverse primers, 2 µl of DNA template and 9.5 µl nuclease-free water. The PCR program consisted of the following steps: initial denaturation at 94 °C for 3 minutes, 35 cycles at 94 °C for 45 sec, 50 °C for 1 minute, 72 °C for 1 minute 30 second and final extension step at 72 °C for 10 minutes. Visual inspection of the PCR products was performed on a 1% agarose gel stained with SYBR safe (Life Technologies, CA, USA). All samples were amplified in triplicate and combined prior to PCR purification with QIAquick PCR Purification kit (Qiagen, Valencia, CA, USA) following the manufacturer’s instruction. Purified products were pooled together in equal concentration for submission to the UC Davis Genome Center, DNA Technology Core Facility. The 16S rDNA libraries were then sequenced for 250 bp paired-end reads on the Illumina MiSeq platform. The raw sequencing data is available at the European Nucleotide Archive (http://www.ebi.ac.uk/ena) under the EBI accession: ERP108716. For the metabolic profiling, frozen samples of the cecal content were submitted to the NIH West Coast Metabolomic Center, UC Davis.

### Microbiome data processing and analysis

The QIIME version 1.9.1^36^ pipeline was used to de-multiplexed, quality-filter and analyze the multiplexed sequence reads obtained as described previously^11^. Briefly, OTU were clustered against GreenGenes 16S rRNA reference database version 13_8 at 97% identity. Both alpha (Chao1 richness & Shannon’s diversity) and beta diversity metrics (weighted uniFrac PCoA plot) were used to analyze the overall microbiota profile. Alpha diversity metrics were evaluated with Mann-Whitney *U* test while ANOISM with 999 permutations was performed with reported *r* and *p* value for beta-diversity with weighted unifrac PCoA^36^. Analysis of the relative abundance of the microbial community at family phylogenetic level was performed with linear discriminant analysis (LDA) effect size analysis (LEfSe) in the Galaxy framework^37,38^. The LEfSe built-in workflow performs three steps to detect features that are statistically significant among the biological class of interest. It first uses the non-parametric Kruskal-Wallis sum rank test to analyze all features and detect significant differential abundance features in classes of interest. Next, Wilcoxon rank-sum test performs pairwise comparison between the subclasses within different classes to determine whether it follows the class level trend. As the last step, Linear Discriminant Analysis (LDA) is performed to estimate the effect size of differentially abundant features with respect to classes, which are consistent with the subclass grouping within classes and ranked them accordingly^39^. The alpha value of 0.05 and an effect size threshold of 2 were used to identify the significant biomarkers in each group.

### PICRUSt functional metagenomic prediction

Metagenomic functional features of the microbial community were predicted using phylogenetic investigation communities by reconstruction of unobserved states (PICRUSt)^17^. Closed-reference OTU picks from QIIME were aligned to search for 16S sequences matching against the green genes reference OTUs at 97% identity. Generated biom file was normalized by dividing each OTU by known/predicted 16S copy number abundance and used for metagenomic prediction with PICRUSt where Kyoto Encyclopedia of Genes and Genomes (KEGG) genes & pathway abundance for each individual sample were generated. Data were then analyzed with Statistical Analysis of Taxonomic and Functional Profiles (STAMP) version 2.1.3^18^. Differentially represented functional pathways (level 3 in hierarchy, representing KEGG pathways) between the two conditions (presented in extended error bar plots) were analyzed with two-sided Welch’s t-test on every pair of means where p <0.05 was considered significant. Confidence intervals of 95% were obtained by inverting the Welch’s tests.

### Metabolite data analysis

Metabolites data sets from a total of 39 samples (20 non-infected and 19 SE-infected chicks) were analyzed with MetaboAnalyst 3.0^19–24^ (http://www.metaboanalyst.ca/faces/home.xhtml) for statistical analysis. Peak intensity raw data set were filtered first with interquantile range. Data were then log transformed and normalized by auto scaling. Both univariate and multivariate analyses were performed on the data set to differentiate the metabolite profiles of non-infected and SE-infected group as well as to identify potential biomarkers associated with SE-infection. For univariate analysis, significant metabolites that were differentially represented in the two groups were analyzed with volcano plots that incorporated both fold change threshold of 1.5 and t-test, with an FDR adjusted p-value ≤ 0.1. Principal component analysis (PCA) was performed with unsupervised multivariate analysis. For supervised multivariate analysis, partial-least squares discrimination analysis (PLS-DA) was performed and validated using R^2^ (close to 1) and Q^2^ (>0.5) threshold value based on leave-one-out cross validation. Higher score of both R^2^ (closer to 1) and Q^2^ >0.5 were used to confirm the significant & predictive power of the PLS-DA model. Contribution of the biomarker metabolites to the group separation in the PLS-DA model was ranked using the variable importance in projection (VIP) score >1. Hierarchical clustering with heatmap analysis based on student t-test was used to identify differential expression of the top 25 metabolites between the two conditions. Significance analysis for microarrays (SAM) method (designed to address the false discovery rate, FDR) was utilized to identify the top discriminant metabolites with q-value ≤ 0.1. Candidate biomarker metabolites were then selected for differential regulation of metabolite pathway analysis using the combined statistical criteria of FDR adjusted p-value <0.1 & fold change threshold of 1.5 (Volcano plot analysis), q-value ≤ 0.1 (SAM), and VIP score >1.0 (PLSDA). Up-regulation of identified biomarker metabolites associated with SE-infection were mapped to the gallus gallus (chicken) pathway library with pathway analysis algorithms of hypergeometric test and relative-betweenness centrality (pathway topology analysis). Likewise, down-regulation of the pathway with an associated reduced level of identified metabolites were performed in the same manner.
